# DNMT3b promotes proliferation and invasion by mediating HOPX DNA methylation in lung cancer

**DOI:** 10.1016/j.isci.2025.114347

**Published:** 2025-12-05

**Authors:** Kelei Guan, Songfeng Zhao, Guizhen Zhang, Yun Wang, Dongdong Song, Yanxia Ding

**Affiliations:** 1 Department of Pharmacy, the First Affiliated Hospital of Zhengzhou University, Zhengzhou 450000 china; 2 Precision Medicine Center, the First Affiliated Hospital of Zhengzhou University, Zhengzhou 450000 china; 3 Department of Rheumatology and Immunology, the First Affiliated Hospital of Zhengzhou University, Zhengzhou 450000 china

**Keywords:** biological sciences, epigenetics, cell biology, cancer

## Abstract

DNMT3B is an important DNA methyltransferase related with unfavorable outcomes for cancer patients. DNMT3B can promote the progression of multifarious malignant tumors. Nevertheless, the functional mechanisms through which DNMT3B promotes the malignant progression of lung cancer remain incompletely understood and require further investigation. In this study, we demonstrated that DNMT3B promoted proliferation, migration, and invasion of lung cancer cells *in vitro* and facilitated tumor growth *in vivo* in xenograft models. Mechanistically, DNMT3B could downregulate HOPX expression through DNA methylation. Consistently, the DNMT inhibitor (SGI-1027) could significantly upregulate HOPX expression level. High HOPX expression effectively suppressed the proliferation, migration, and invasion of lung cancer cells. In contrast, HOPX knockdown partially recovered the malignant phenotypes of lung cancer cells treated with SGI-1027 or si-DNMT3B. In conclusion, these findings provide a rationale for targeting DNMT3B-mediated HOPX DNA methylation and identify crucial molecular targets for lung cancer therapy.

## Highlights

• DNMT3B promotes proliferation, migration, and invasion of lung cancer cells
• DNMT3B downregulates HOPX expression through DNA methylation
• DNMT3B-mediated HOPX silencing provides a promising therapeutic target for lung cancer

## Graphical abstract

**Figure:**
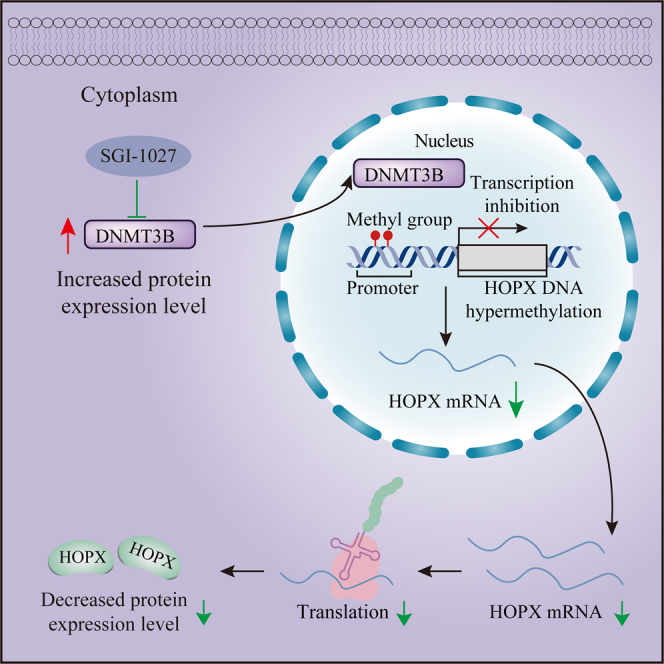

## Introduction

Lung cancer is one of the deadliest malignant tumors globally and the most commonly diagnosed cancer, accounting for 18% of all cases.^1^^,^^2^ Lung cancer remains the leading cause of cancer-related mortality, accounting for 23% of all cancer-related deaths.^3^ Despite advances in treatment strategies—including surgery, radiotherapy, chemotherapy, and targeted therapy—the prognosis remains poor, with a 5-year survival rate of approximately16.8%.^4^ There are many reasons for the low 5-year survival rate, including delayed diagnoses (often at advanced stages), nonspecific early symptoms, and limited access to targeted therapies due to undetected molecular markers.^5^ Several studies have reported that epigenetic mechanisms—including DNA methylation, chromatin remodeling, histone modifications, and noncoding RNA regulation—play a crucial role in cancer development and progression. Among these, DNA methylation is particularly critical in lung cancer pathogenesis.^6^^,^^7^ The precise mechanisms through which DNA methylation promotes lung cancer progression require further investigation.

DNA methylation plays an important role in regulating gene expression, genomic imprinting, and X chromosome inactivation.^8^ Tumor suppressor genes with CpG-rich promoters are inactivated by promoter hypermethylation during tumorigenesis.^9^ DNA methylation at the 5′-cytosine of CpG sites is facilitated by three cytosine DNA methyltransferase enzymes including DNMT1, DNMT3A, and DNMT3B. DNMT1 is a maintenance methyltransferase that plays a crucial role in establishing normal methylation patterns during embryogenesis.^10^^,^^11^ DNMT3A and DNMT3B play essential roles in establishing DNA methylation patterns.^12^ Furthermore, DNMTs (DNMT1/3A/3B) and ten-eleven translocation (TET) enzymes (TET1/2/3) reciprocally regulate 5-mC and 5-hmC levels, establishing an epigenetic landscape critical for gene expression control.^13^ The dysregulation of these mechanisms is related to progression of cancer. For example, it was reported that DNMT3A and DNMT3B contributed to epigenetic reprogramming in multiple cancers. MicroRNA-30a-3p was shown to suppress lung cancer progression by targeting DNA methyltransferase 3A (DNMT3A) through the PI3K/AKT pathway. Meanwhile, Ji Yong et al. found that methyltransferase DNMT3B promoted colorectal cancer cell proliferation by inhibiting PLCG2.^14^^,^^15^ Similarly, Wu Qianbiao et al. discovered that DNMT3B-mediated SPAG6 promoter hypermethylation affected lung squamous cell carcinoma development through the JAK/STAT pathway.^16^ However, it needs to be further investigated whether there are additional mechanisms by which DNMT3B influences the progression and prognosis of lung cancer.

Homeodomain-only protein homeobox (HOPX) is the protein with the smallest homology domain, whose gene is situated on human chromosome 4, and composed of seven exons.^17^^,^^18^ As a transcription factor, HOPX exhibits widespread expression across diverse tissues and organs.^19^ Importantly, HOPX functions as a crucial tumor suppressor in various cancers. Epigenetic silencing of HOPX through promoter methylation was frequently observed and showed cancer specificity in human malignancies. Some studies have demonstrated that HOPX is related to cancer progression and cancer patient prognosis. For instance, Liang Huagang et al. confirmed that microRNA-421 promoted the progression of non-small cell lung cancer by targeting HOPX and regulating the Wnt/β-catenin signaling pathway. Similarly, Yosuke Ooizumi et al. demonstrated that epigenetic silencing of HOPX was critically involved in aggressive phenotypes and patient prognosis in papillary thyroid cancer.^20^^,^^21^ Nevertheless, the underlying molecular mechanisms of HOPX epigenetic silencing in lung cancer remain unclear and require further exploration.

DNMT3B participates in epigenetic reprogramming in multiple cancers. Although epigenetic silencing of HOPX has been widely observed in numerous cancers, whether DNMT3B mediates this silencing in lung cancer remains to be further explored. In this study, we demonstrated that DNMT3B was significantly upregulated in lung cancer. Functionally, DNMT3B promoted the proliferation, migration, and invasion of lung cancer cell. Mechanistically, we found that DNMT3B repressed HOPX expression through DNA methylation, which in turn affected the proliferation, migration, and invasion of lung cancer cells. Collectively, our current research illuminates a molecular mechanism of the DNMT3B-mediated-HOPX DNA methylation axis and provides molecule targets for lung cancer therapy.

## Results

### Elevated expression of DNMT3B in lung cancer tissues is related with poor survival

**Figure 1:**
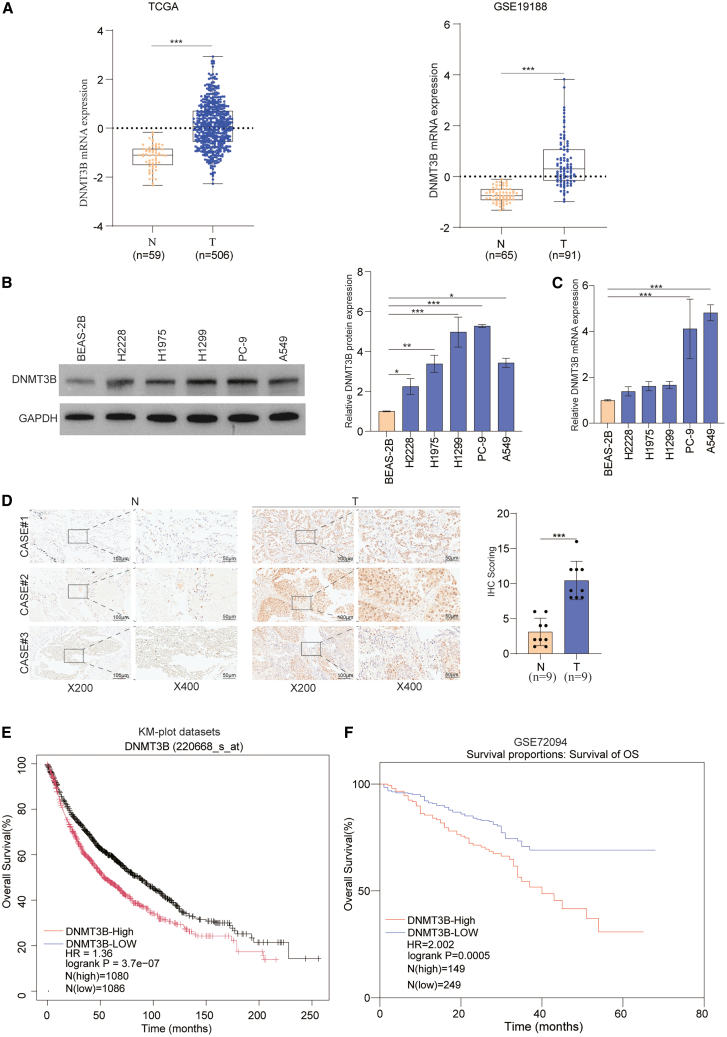
Upregulated expression of DNMT3B was associated with poor progression in lung cancer tissue (A) Expression of DNMT3B mRNA in lung cancer tissue from the TCGA and GEO datasets. (B and C) Protein and mRNA expression levels of DNMT3B were compared between normal and cancerous lung cells. (D) IHC analysis of DNMT3B in lung cancer TMA cohorts. Scale bars: 100 μm/50 μm. (E and F) Kaplan-Meier survival analysis by DNMT3B expression status in lung cancer. Data are presented as the mean ± SD of at least 3 independent experiments; ∗*p* < 0.05, ∗∗*p* < 0.01, ∗∗∗*p* < 0.001.

To investigate the expression status and clinical significance of DNMT3B in lung cancer, we first explored its expression profile by utilizing The Cancer Genome Atlas (TCGA) and GEO datasets. We discovered that DNMT3B expression was markedly upregulated in lung cancer tissues (Figure 1A). Furthermore, higher levels of DNMT3B were expressed in lung cancer cell line than in normal bronchial epithelial cells (BEAS-2B) (Figures 1B and 1C). Immunohistochemical (IHC) staining was performed on samples from tissue microarray (TMA) cohorts, which further displayed the high expression of DNMT3B in lung cancer tissues (Figure 1D). Moreover, we assessed the clinical significance of DNMT3B using Kaplan-Meier analysis of the km-plot datasets (220668) and the GSE72094 dataset and found that high expression of DNMT3B was associated with poor survival of lung cancer patients (Figures 1E and 1F). Together, these results demonstrate that DNMT3B is highly expressed in lung cancer and is closely correlated with a poor prognosis of patients.

### Downregulation of DNMT3B suppresses the proliferation of lung cancer cells *in vitro*

**Figure 2:**
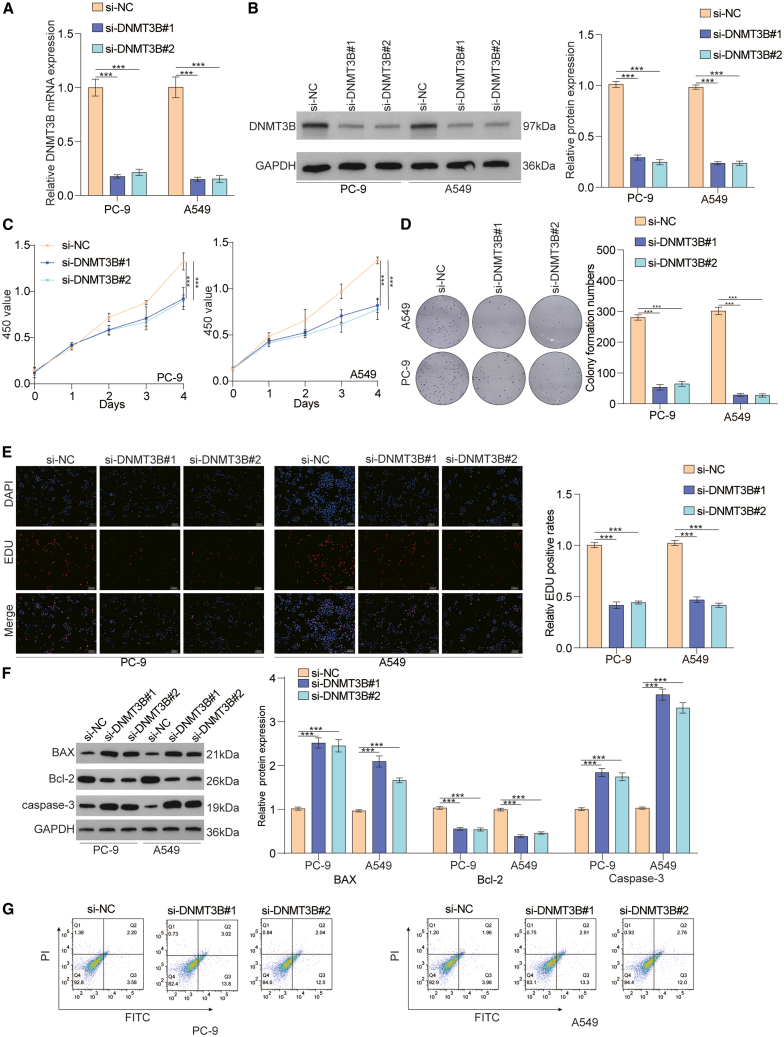
Downregulation of DNMT3B inhibited proliferation of lung cancer cells *in vitro* (A and B) The efficiency of DNMT3B knockdown in lung cancer cells was measured through reverse-transcription PCR (RT-PCR) and western blotting. (C–E) The proliferation of DNMT3B-deficient cells was assessed using CCK-8, colony formation, and EdU assays. Scale bar, 100 μm. (F) The protein expression status of genes related with cell apoptosis during DNMT3B knockdown was determined via western blot. (G) Annexin V and propidium iodide (PI) staining for PC-9 and A549 cells treated with si-NC or si-DNMT3B. Data are presented as the mean ± SD of at least 3 independent experiments; ∗*p* < 0.05, ∗∗*p* < 0.01, ∗∗∗*p* < 0.001.

We subsequently explored the tumorigenic role of DNMT3B in lung cancer cells. We used a DNMT3B-specific small interfering RNA (siRNA) to effectively silence its expression (Figures 2A and 2B). Cell proliferation assay showed that the downregulation of DNMT3B significantly decreased the proliferation of lung cancer cells (Figures 2C–2E). In addition, we examined the effect of DNMT3B on the expression of genes associated with cell apoptosis and observed that the downregulation of DNMT3B increased the expression of BAX and caspase3 in lung cancer cells (Figure 2F). Subsequently, we further confirmed that downregulation of DNMT3B markedly promoted the apoptosis of lung cancer cells by flow cytometry analysis (Figure 2G). In summary, our experimental data have shown that downregulation of DNMT3B inhibits the proliferation of lung cancer cells.

### Downregulation of DNMT3B inhibits the migration and invasion of lung cancer cells *in vitro*

**Figure 3:**
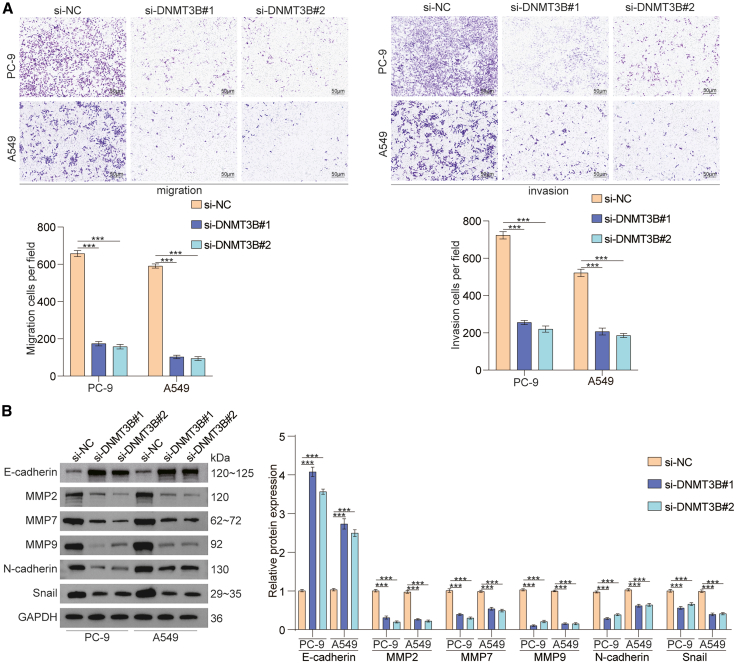
Downregulation of DNMT3B suppressed migration and invasion of lung cancer cells *in vitro* (A) Transwell assays of lung cancer cells with DNMT3B knockdown. Scale bars, 50 μm. (B) The protein expression status of genes related with EMT in lung cancer cells with DNMT3B knockdown were checked through western blotting. Data are presented as the mean ± SD of at least 3 independent experiments; ∗*p* < 0.05, ∗∗*p* < 0.01, ∗∗∗*p* < 0.001.

To evaluate the effect of DNMT3B on lung cancer migration and invasion, the transwell assay was performed. First, we found that the knockdown expression of DNMT3B could inhibit the migratory and invasive abilities of lung cancer cells (Figure 3A). Second, we explored the influence of DNMT3B on the expression of genes associated with epithelial-mesenchymal transition (EMT) and found that the downregulation of DNMT3B markedly decreased the expression of MMP2, MMP7, MMP9, SNAIL, and N-cadherin in lung cancer cells *in vitro* (Figure 3B). These results show that downregulation of DNMT3B reduces the migratory and invasive capacities of lung cancer cells.

### Upregulation of DNMT3B promotes the migration and invasion of lung cancer cells *in vitro*

**Figure 4:**
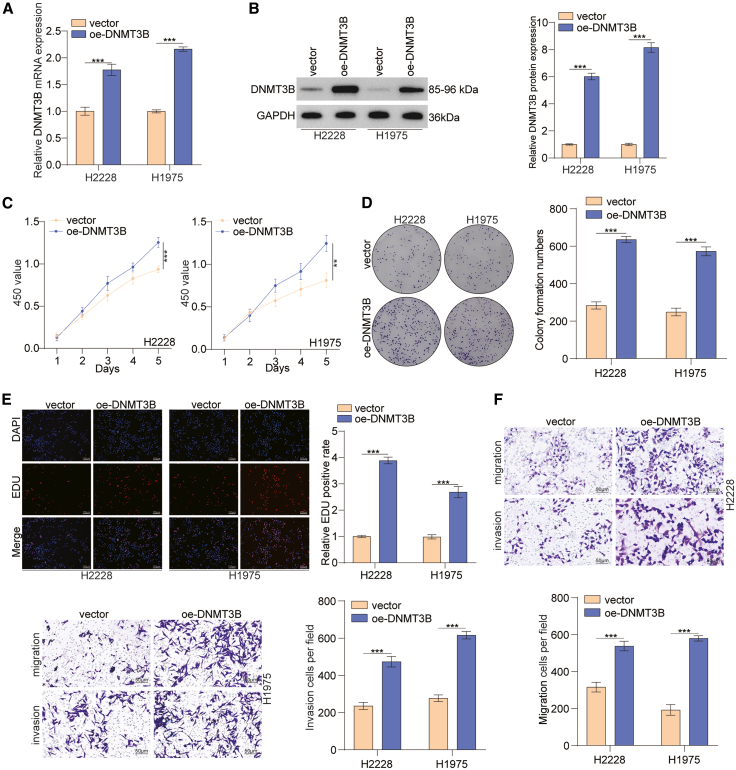
Upregulation of DNMT3B promoted proliferation, migration, and invasion of lung cancer cells *in vitro* (A and B) The efficiency of DNMT3B overexpression in lung cancer cells was measured through RT-PCR and western blot. (C–E) The proliferation of DNMT3B-overexpressing cells was evaluated by CCK-8, colony formation, and EdU assays. Scale bars, 150 μm. (F) Transwell assays of lung cancer cells with DNMT3B overexpression. Scale bars, 50 μm. Data are presented as the mean ± SD of at least 3 independent experiments; ∗*p* < 0.05, ∗∗*p* < 0.01, ∗∗∗*p* < 0.001.

In order to investigate the influence of DNMT3B overexpression on lung cancer cells, we used a DNMT3B-overexpression plasmid to accomplish the efficient overexpression of DNMT3B in lung cancer cells (Figures 4A and 4B). Then, the proliferative ability of DNMT3B-overexpression lung cancer cells was evaluated by CCK-8, colony formation, and 5-ethynyl-2′-deoxyuridine (EdU) assays. Consistent with the previous results, DNMT3B overexpression signally promoted the proliferation of lung cancer cells (Figures 4C–4E). In addition, transwell assays showed that DNMT3B overexpression markedly elevated the migratory and invasive capabilities of lung cancer cells (Figure 4F). In conclusion, overexpression of DNMT3B enhances the migratory and invasive capabilities of lung cancer cells.

### DNMT3B promotes lung cancer growth *in vivo*

**Figure 5:**
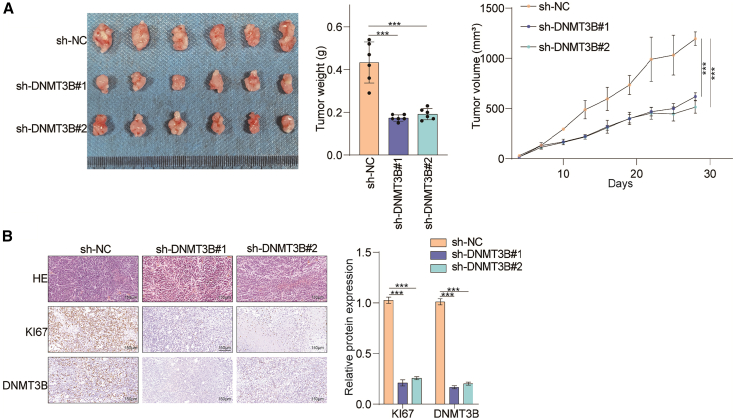
DNMT3B facilitates the growth of xenograft tumor *in vivo* (A) Morphological images of an orthotopic-xenograft mouse model was generated by subcutaneously injecting A549 cells with DNMT3B knockdown, and tumor volume and weight were measured. (B) Hematoxylin and eosin (H&E) staining and IHC staining of DNMT3B and Ki67 in tumor sections. Scale bars, 150 μm. Data are presented as the mean ± SD of at least 3 independent experiments; ∗*p* < 0.05, ∗∗*p* < 0.01, ∗∗∗*p* < 0.001.

We deeply investigated the oncogenic role of DNMT3B *in vivo*. We generated an orthotopic-xenograft mouse model by subcutaneously injecting lung cancer cells infected with lentivirus containing either sh-DNMT3B or sh-NC. Consistent with the *in vitro* results, we observed that tumor weight and volume decreased remarkably in the DNMT3B knockdown groups compared to the control group (Figure 5A). Moreover, IHC analysis demonstrated that the DNMT3B knockdown groups exhibited weaker Ki-67 staining than the control group (Figure 5B). These findings indicate that DNMT3B significantly facilitates lung cancer growth *in vivo*.

### DNMT3B expression is negatively correlated with HOPX expression and mediates the DNA methylation of HOPX

**Figure 6:**
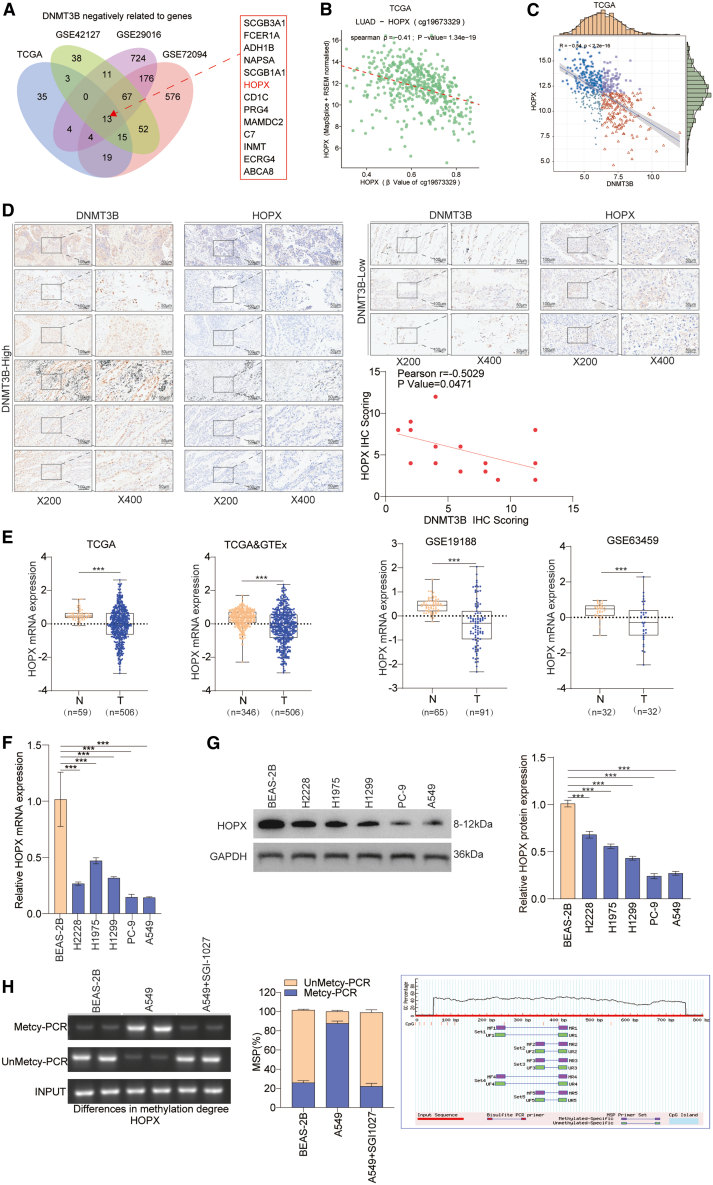
DNMT3B was negatively correlated with the expression of HOPX (A) Genes negatively correlated with DNMT3B were identified through Venny analysis (https://www.omicstudio.cn/tool). (B) Negative correlation between cg19673329 methylation and HOPX expression in TCGA cohort (*ρ* = −0.41, *p* < 0.001). (C) Negative correlation between DNMT3B and HOPX expression in TCGA cohort (*r* = −0.54, *p* < 0.001). (D) The correlation of DNMT3B and HOPX expression was determined by TMA analysis (*r* = −0.5029, *p* < 0.05). Scale bars, 100 μm/50 μm. (E) HOPX expression was assessed in lung cancer tissues and adjacent non-cancerous tissues using the TCGA, GTEx, and GEO datasets. (F and G) HOPX mRNA and protein expression levels were assessed in various lung cancer cells. (H) DNA methylation levels in different lung cancer cells were checked by methylation-specific PCR. Data are presented as the mean ± SD of at least 3 independent experiments; ∗*p* < 0.05, ∗∗*p* < 0.01, ∗∗∗*p* < 0.001.

Given that DNMT3B is an important DNA methyltransferase enzyme, we deeply investigated the functional targets of DNMT3B as well. First, we performed a correlation analysis of DNMT3B and other genes by utilizing TCGA and three GEO datasets and found that 13 genes showed a negative correlation with DNMT3B (Figure 6A). We also detected that the methylation status of the HOPX locus (cg19673329) was negatively correlated with HOPX expression in lung cancer. Furthermore, DNMT3B expression was negatively correlated with HOPX expression in the TCGA dataset (Figures 6B and 6C). Consistent with previous results, TMA analysis revealed a strong negative correlation between HOPX and DNMT3B expression levels (*r* = −0.5029, *p* < 0.05) (Figure 6D). Second, we found that HOPX expression was significantly lower in lung cancer tissues compared to adjacent non-cancerous tissues across the TCGA, GTEx, and GEO datasets (Figure 6E). Moreover, our data suggest that lung cancer cell lines have lower mRNA and protein levels of HOPX compared to the normal bronchial epithelial cells (BEAS-2B) (Figures 6F and 6G). We further analyzed HOPX methylation levels in BEAS-2B and lung cancer cell lines using methylation-specific PCR. We found that the methylation level of HOPX was lower in BEAS-2B cells than A549 cells and DNMT inhibitor (SGI-1027) reduced the methylation level of HOPX in A549 cells (Figure 6H). Taken together, these findings suggest that DNMT3B mediates methylation of HOPX.

### HOPX is identified as a critical downstream regulatory target of DNMT3B in lung cancer

**Figure 7:**
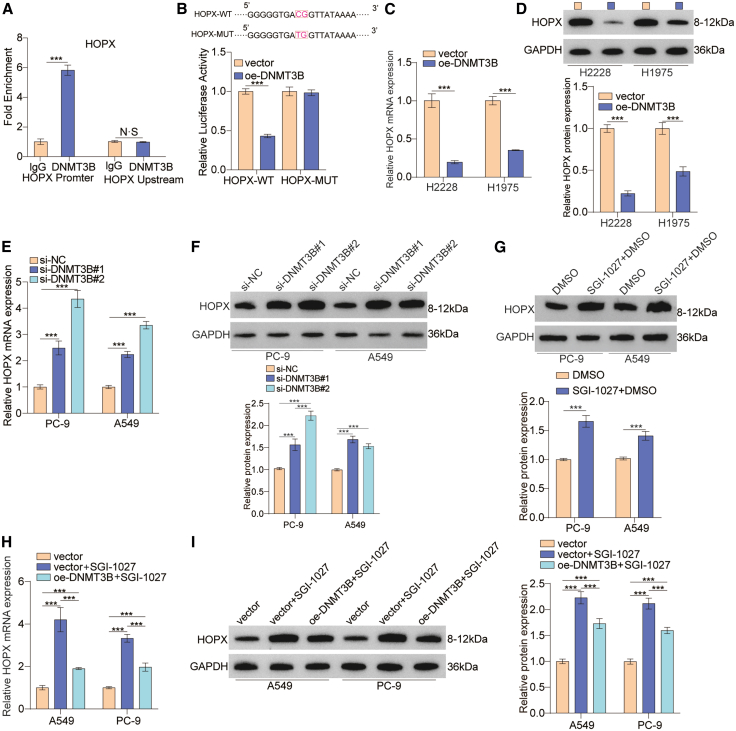
HOPX was identified as a downstream regulatory target of DNMT3B (A) Fold enrichment of DNMT3B in the promoter and upstream region of HOPX gene was checked by ChIP-qPCR. (B) Luciferase reporter assays were performed by co-transfecting the wild-type HOPX promoter or the mutant-type HOPX promoter with DNMT3B overexpression vector or blank vector in 293T cells. (C and D) HOPX mRNA and protein expression levels were measured in H2228 and H1975 cells treated with oe-DNMT3B or vector. (E and F) The mRNA and protein expression levels of HOPX were examined in lung cancer cells treated with si-DNMT3B or si-NC. (G) The protein expression status of HOPX was checked in lung cancer cells treated with SGI-1027. (H and I) Rescue experiments examined the mRNA and protein expression levels of HOPX in A549 and PC-9 cells treated with vector, vector+SGI-1027, or oe-DNMT3B+SGI-1027. Data are presented as the mean ± SD of at least 3 independent experiments; ∗*p* < 0.05, ∗∗*p* < 0.01, ∗∗∗*p* < 0.001.

For investigating the functional downstream targets of DNMT3B, we further explored the interaction between DNMT3B and HOPX. Chromatin immunoprecipitation (ChIP)-qPCR revealed that DNMT3B bound to the promoter region of HOPX (Figure 7A). Luciferase reporter assays showed that DNMT3B overexpression inhibited wild-type HOPX promoter luciferase activity in 293T cells (Figure 7B). These results suggest that DNMT3B regulates HOPX expression via DNA methylation. In addition, we checked the expression level of HOPX in H2228 and H1975 cells treated with oe-DNMT3B and found that the expression of HOPX was downregulated (Figures 7C and 7D). Next, we also examined the expression level of HOPX in PC-9 and A549 cells treated with si-DNMT3B or the DNMT inhibitor SGI-1027. Interestingly, the cells treated with si-DNMT3B or SGI-1027 exhibited significantly higher HOPX expression than control groups (Figures 7E–7G). For further verification, we performed rescue experiments by re-expressing DNMT3B in lung cancer cells treated with SGI-1027. Notably, HOPX expression was partially downregulated in cells treated with both oe-DNMT3B and SGI-1027 compared to SGI-1027-only controls (Figures 7H and 7I). Collectively, these results indicate that HOPX is a regulatory target of DNMT3B in lung cancer.

### Overexpression of HOPX repressed proliferation, migration, and invasion of lung cancer cells *in vitro*

**Figure 8:**
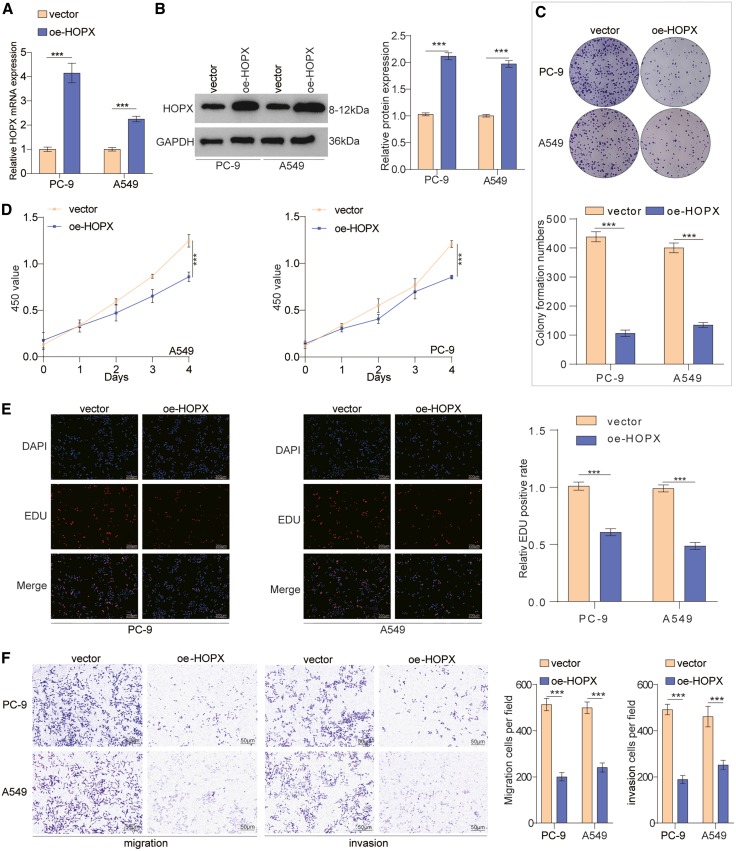
HOPX overexpression repressed progression, migration, and invasion of lung cancer cells (A and B) The efficiency of HOPX overexpression was validated. (C–E) The proliferation ability of lung cancer cells treated with oe-HOPX or control vectors were assessed by the CCK8, colony formation, and EdU assays. Scale bar: 200 μm. (F) Transwell assay evaluating the migratory and invasive ability of lung cancer cells treated with oe-HOPX or control vectors. Scale bars, 50 μm. Data are presented as the mean ± SD of at least 3 independent experiments; ∗*p* < 0.05, ∗∗*p* < 0.01, ∗∗∗*p* < 0.001.

Considering that HOPX was identified as an important regulated target of DNMT3B, we further investigated the effects of HOPX overexpression in lung cancer. We utilized a HOPX-overexpressing plasmid to achieve efficient HOPX overexpression in lung cancer cells (Figures 8A and 8B). Overexpression of HOPX markedly suppressed cell proliferation (Figures 8C–8E). Next, to investigate the function of HOPX in lung cancer metastasis, we checked the migration and invasion ability of lung cancer cell. Interestingly, we noticed that compared with the control groups, overexpression of HOPX significantly weakened the capabilities of migration and invasion in lung cancer cells (Figure 8F). These results suggest that HOPX represses the proliferation and metastasis of lung cancer cells *in vitro*.

### Knockdown of HOPX restored the proliferative, migratory, and invasive abilities of lung cancer cells treated with SGI-1027 or si-DNMT3B *in vitro*

**Figure 9:**
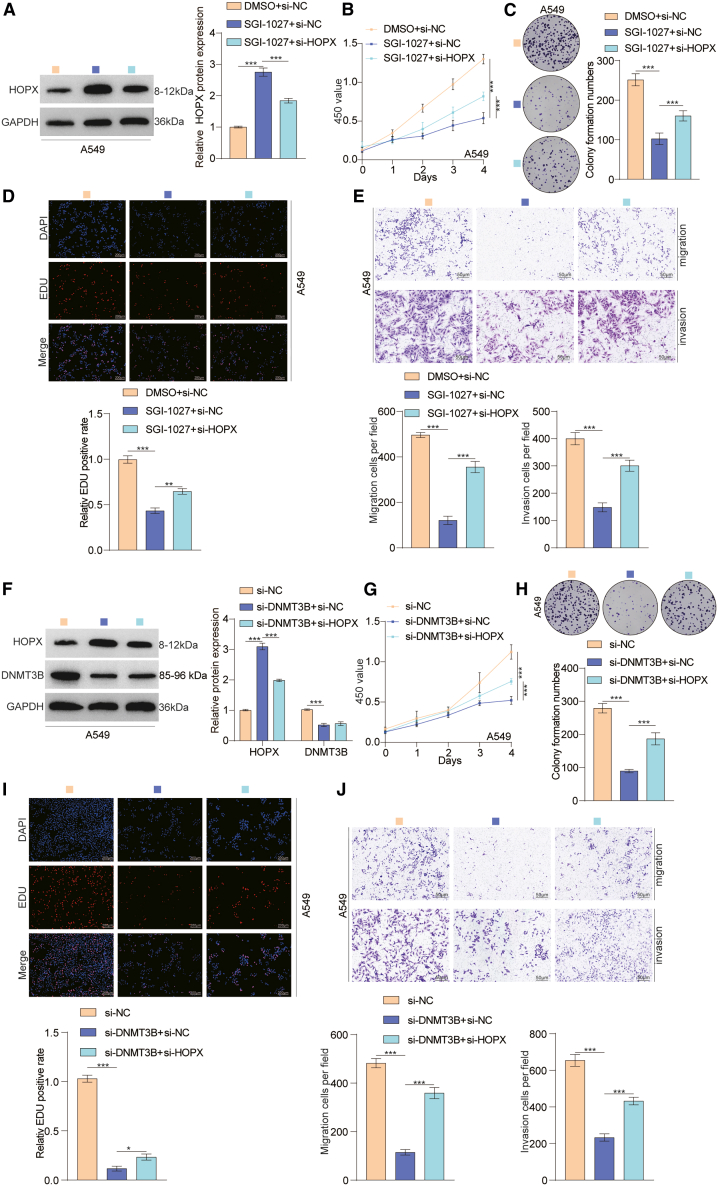
Downregulation of HOPX facilitates proliferation, migration, and invasion of lung cancer cells treated with SGI-1027 or si-DNMT3B (A) The expression level of HOPX was measured by western blot in A549 cells treated with DMSO+si-NC, SGI-1027+si-NC, or SGI-1027+si-HOPX. (B–D) CCK8, colony formation, and EdU assays checked the cell proliferation ability of HOXP-knockdown and control groups in A549 cells treated with SGI-1027. Scale bar:200 μm. (E) Transwell assay evaluated the migratory and invasive abilities of A549 cells treated with DMSO+si-NC, SGI-1027+si-NC, or SGI-1027+si-HOPX. Scale bars, 50 μm. (F) HOPX and DNMT3B protein levels were measured through western blot in A549 cells treated with si-NC, si-DNMT3B+si-NC, or si-DNMT3B+si-HOPX. (G–I) CCK8, colony formation, and EdU assays checked the cell proliferation ability of HOXP-knockdown and control groups in A549 cells treated with si-DNMT3B. Scale bars, 200 μm. (J) Transwell assay evaluated the migratory and invasive abilities of A549 cells treated with si-NC, si-DNMT3B+si-NC, or si-DNMT3B+si-HOPX. Scale bar, 50 μm. Data are presented as the mean ± SD of at least 3 independent experiments; ∗*p* < 0.05, ∗∗*p* < 0.01, ∗∗∗*p* < 0.001.

**Figure 10:**
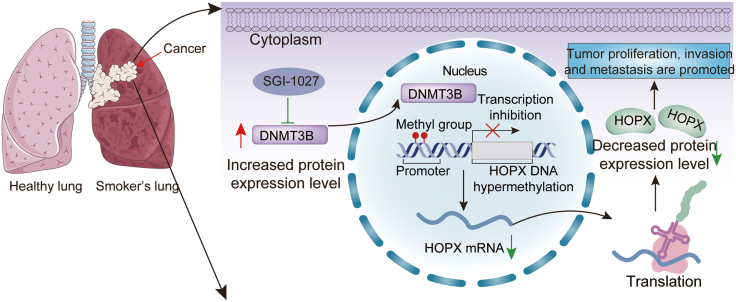
The schematic figure showed the mechanism of DNMT3B mediating HOPX gene methylation (Synopsis image was performed from Servier Medical Art, http://smart.servier.com).

Our previous findings indicated that the overexpression of HOPX remarkably suppressed the proliferative, migratory, and invasive capabilities of lung cancer cells. Consequently, we proceeded to explore whether HOPX silence could partially restore the progressive ability of lung cancer cells. We utilized HOPX-specific siRNA to efficiently knockdown the expression of HOPX in lung cancer cells treated with a DNMT inhibitor (SGI-1027) or si-DNMT3B (Figures 9A and 9F). We observed that the downregulation of HOPX partly restored the proliferation of lung cancer cells (Figures 9B–9D and 9G–9I). Moreover, to explore the function of HOPX in lung cancer metastasis, we performed cell migration and invasion assays. Compared to control cells, the migratory and invasive abilities of lung cancer cells were partly recovered following HOPX knockdown (Figures 9E and 9J). In conclusion, these results suggest that DNMT3B-mediated HOPX DNA methylation promotes the development of lung cancer. Figure 10 illustrates the functional landscape of the DNMT3B/HOPX DNA methylation axis in driving lung cancer progression.

## Discussion

Numerous studies have shown that DNA methylation is one of the most consistent epigenetic alterations in various human cancers.^22^^,^^23^ DNA methyltransferases are significantly associated with the transformation and progression of human cancers by mediating the hypermethylation of tumor suppressor factors.^24^ As one of the three vital DNA methyltransferases, DNMT3B is significantly associated with adverse clinical outcomes in cancer patients.^10^^,^^25^ Many investigations have indicated that DNMT3B promotes the development and metastasis of various malignant tumors. For example, Zhang Haibin et al. found that DNMT3B was widely expressed in a variety of hematological cells and its expression was altered in each type of leukemia, which was closely associated with the pathogenesis, progression, and prognosis of leukemia.^26^ Ji Yong et al. further demonstrated that methyltransferase DNMT3B promoted colorectal cancer cell proliferation by inhibiting PLCG2.^15^ Another study discovered that DNMT3B had a high protein expression level in bladder cancer tissues, and it was correlated with poor clinical prognosis.^27^ Meanwhile, a contrary report by Lorenzo Rinaldi et al. demonstrated that Dnmt3a and DNMT3B protected the epidermis against tumorigenesis and that squamous cell carcinomas were sensitive to PPAR-γ inhibition. Consistent with the discovered oncogenic role of DNMT3B, we found that DNMT3B was upregulated in lung cancer and associated with poor outcomes of lung cancer patients. High expression of DNMT3B facilitated the proliferation, migration, and invasion of lung cancer cells. In addition to this, we discovered that DNMT3B repressed the expression of genes associated with cell apoptosis, and promoted the expression of genes related with EMT.^28^^,^^29^ These findings show that DNMT3B is vital for lung cancer progression and closely related to lung cancer prognosis. Thus, DNMT3B can be regarded as a potential molecule target of lung cancer therapy.

Additionally, we further investigated the mechanisms through which DNMT3B promotes the progression and metastasis of lung cancer. Herein, we demonstrated that DNMT3B downregulates the expression of HOPX via DNA methylation and then promotes the proliferation, migration, and invasion of lung cancer cells. Multiple studies were in agreement with our research. For example, Wu Hao et al. discovered that DNMT3B and TET1 regulated breast cancer through the Hippo signaling pathway by mediating DNA methylation of the large tumor suppressor gene 1 (LATS1).^30^ Similarly, Liu Yan et al. found that methylation of PCDH17 mediated by DNMT3B affected the progression of hepatocellular carcinoma through EMT.^31^ In addition, it had been demonstrated that DNMT3B affected colorectal cancer advancement by mediating DNA hypermethylation of FLI.^32^ A study revealed that DNMT3B-mediated FAM111B methylation promoted the glycolysis, growth, and metastasis of papillary thyroid tumor.^33^ These results indicate that DNMT3B is an important DNA methyltransferase enzyme and participates in target gene methylation, which promotes the progression of a variety of cancers. Thus, the mechanism by which DNMT3B regulates the expression of target genes via DNA methylation is able to provide ideas for advancing cancer treatment strategies and tumor-suppressive function.^18^ However, there were the other mechanisms by which DNMT3B regulated lung cancer development. For example, Bo Yang et al. found that DNMT3B regulated the proliferation of A549 cell through the microRNA-152-3p/NCAM_1_ pathway.^34^ It was reported by Yang Pingshan et al. that miR-203a-3p-DNMT3B feedback loop facilitates non-small cell lung cancer progression.^35^ More potential mechanisms by which DNMT3B regulated lung cancer progression still require further investigation.

The role of HOPX in tumor growth has been demonstrated by a lot of reports. Bourque et al. found that HOPX mediated the growth of some tumors.^36^ It also was discovered that hypermethylation of the HOPX locus led to the down-expression of itself, which was related to metastatic cancer, including papillary thyroid cancer.^21^ Meanwhile, it was demonstrated by Ren that HOPX regulated the promoter silencing of SNAIL in tumors, which was a transcription factor that initiates EMT.^37^ These reports were in accordance with our findings. In this research, we detected that the HOPX expression was lower in lung cancer cells. The over-expression of HOPX was able to suppress the proliferation, invasion, and migration abilities of lung cancer cells. These findings indicate that HOPX, as a crucial suppressor gene of tumor, has an important impact on the proliferation, migration, and invasion of lung cancer cells.^38^^,^^39^^,^^40^ HOPX can be used as a molecular target for lung cancer treatment.

We also observed that SGI-1027 affected the oncogenic function of DNMT3B/HOPX/DNA methylation axis, thereby suppressing proliferation, invasion, and migration in lung cancer cells. Similar results have been reported. Qiu Wei et al. found that kaempferol regulated DNA methylation and decreased the level of DNMT3B by accelerating its ubiquitin-proteasome degradation and then inhibited the growth of bladder cancer.^41^ Moreover, it had been confirmed that SGI-1027 effectively hindered the proliferation and dissemination of gastric cancer by downregulating DNMT1 and promoting the expression of RB1.^42^ Thus, DNMT inhibitors could serve as a potential therapeutic strategy for lung cancer treatment. To improve its efficacy and specificity, targeted delivery systems for SGI-1027 could be developed. However, the potential limitations of SGI-1027 as a clinical candidate cannot be ignored, including off-target effects, drug resistance, genomic toxicity, and poor metabolic stability.

In conclusion, our research suggests that DNMT3B regulates HOPX via DNA methylation in lung cancer, thereby promoting cancer progression. These findings not only identify molecular targets for lung cancer treatment but also provide insights into developing targeted delivery systems—such as tumor-specific antibody-conjugated nanoparticles—for DNMT3B-mediated-HOPX DNA methylation axis.

### Limitations of the study

Our study has some limitations. First, the current findings are limited to *in vitro* evidence demonstrating the role of DNMT3B in promoting the migration and invasion of lung cancer cells. The *in vivo* function of DNMT3B in tumor progression remains to be determined. Second, our study has only confirmed that DNMT3B can promote the proliferation and invasion of lung cancer by mediating the DNA methylation of HOPX, and whether DNMT3B relies on other molecular mechanisms remains unknown.

## Resource availability

### Lead contact

Requests for further information and resources should be directed to and will be fulfilled by the lead contact, Yanxia Ding (fccdingyx@zzu.edu.cn).

### Materials availability

This study did not generate new unique reagents.

### Data and code availability

• Source data for all main figures are provided within the manuscript, its supplemental information, or the Figshare public repository (https://doi.org/10.6084/m9.figshare.30405529.v1), as detailed in the key resources table.
• This paper does not report any original code.
• Any additional information required to reanalyze the data reported in this paper is available from the lead contact upon request.

## Acknowledgments

This study was supported by the Projects of Higher Education Institutions in Henan Province (No. 22A310010).

## Author contributions

K.G. and Y.D. performed the study design; K.G., G.Z., and D.S. performed the experiment; W.Y. and G.Z. analyzed data; K.G. wrote the manuscript; K.G., Y.D., and D.S. provided funding; Y.D. and S.Z. revised the manuscript. All authors read and approved the final manuscript.

## Declaration of interests

The authors declare no competing of interests.

## STAR★Methods

### Key resources table

**Table:** 

REAGENT or RESOURCE	SOURCE	IDENTIFIER
**Antibodies**		

DNMT3B	proteintech	26971-1-AP; RRID:AB_2880705
GAPDH	proteintech	60004-1-Ig; RRID:AB_2107436
HOPX	proteintech	11419-1-AP; RRID:AB_10693525
MMP7	proteintech	10374-2-AP; RRID:AB_2144452
E-cadherin	Proteintech	60335-1-Ig; RRID:AB_2881444
N-cadherin	Proteintech	66219-1-Ig; RRID:AB_2881610
MMP9	proteintech	10375-2-AP; RRID:AB_10897178
survivin	proteintech	66495-1-Ig; RRID:AB_2881860
Snai1	proteintech	13099-1-AP; RRID:AB_2191756
MMP-2	proteintech	10373-2-AP; RRID:AB_2250823
BAX	proteintech	50599-2-Ig; RRID:AB_2061561
BCL-2	proteintech	12789-1-AP; RRID:AB_2227948
PCNA	proteintech	10205-2-AP; RRID:AB_2160330
Caspase 3	proteintech	82202-1-RR; RRID:AB_3086469
c-Myc	proteintech	10828-1-AP; RRID:AB_2148585

**Bacterial and virus strains**		

HOPX Lentiviral Overexpression Vector	GeneBiogist	JYSJ-lv035OV-HOPX(h)

**Chemicals, peptides, and recombinant proteins**		

RNA Lysis Buffer	Thermo	15596026
2 x SYBR Green	Servicebio	G3321-15
PBS	Servicebio	714001
RPMI-1640 Medium	Solarbio	31800-500
DMEM Medium	Solarbio	31800-500
Fetal Bovine Serum (FBS)	BIOIND	04001-1ACS
RIPA Lysis Buffer	GENSHARE	JC-PL001
PMSF(100mM)	Solarbio	P0100
Protein Molecular Weight Marker	Biosharp	BL712A
PVDF Membrane	Immobilon	0.45um
BSA	Servicebio	G5001-25G
EDTA	Biosharp	BL727B
Formaldehyde Solution	Macklin	F809702-12X500ml
Transwell Chamber	NEST	725301
SGI-1027	MCE	HY-13962

**Critical commercial assays**		

BCA Protein Assay Kit	Beyotime	P0012
SDS-PAGE Gel Kit	Jingcai Bio	TC-PE022/R
Reverse Transcription Kit	Servicebio	G3330-100
CCK-8 Kit	Cio Biotech	C200-100
EdU Kit	Ribobio	C-10310-1
Cell Lysis Buffer Sample Kit	BPS Bioscience	BPQ-82128
Immunoprecipitation Kit	Beyotime	P2175S
DNA Extraction Kit	Acmec	AC11012-100T
DNA Bisulfite Conversion Kit	Beyotime	D0068S

**Experimental models: Cell lines**		

BEAS-2B	FuHeng	FH0319
H2228	FuHeng	FH0615
H1975	FuHeng	FH0086
H1299	FuHeng	FH0908
PC-9	FuHeng	FH0083

**Oligonucleotides**		

RT-qPCR primers for detection of expression of DNMT3B and HOPX, See Table S3 in supplemental information	This Paper	N/A

**Software and algorithms**		

GraphPad Prism software (version 8.3.0)	GraphPad	N/A
figshare	https://figshare.com	https://doi.org/10.6084/m9.figshare.30405529

### Experimental model and study participant details

#### Animals model

The 4–6-week-old male BALB/c nude mice provided by Skobes Biotechnology Co., Ltd (Henan China) were fed in specific pathogen-free units. At the experimental endpoint, mice were humanely sacrificed by cervical dislocation after anesthesia. Animal studies were approved by The Ethical Review Committees of the First Affiliated Hospital of Zhengzhou University (2024-KY-1721-001). During the animal experimentation process, the researchers adhered to the 3R principles.

For the subcutaneous model, mice were randomly assigned into each group according to the random table method (n=6 per group). 5×10^6^ A549 cells infected with sh-DNMT3B or sh-NC were subcutaneously injected into the right flanks of BALB/c nude mice to generate a xenograft nude mouse model.

### Method details

#### The Cancer Genome Atlas and Gene Expression Omnibus datasets

Gene expression data were acquired from The Cancer Genome Atlas (TCGA; https://portal.gdc.cancer.gov), Gene Expression Omnibus (GEO; https://www.ncbi.nlm.nih.gov/geo/), and Genotype-Tissue Expression (GTEx; https://gtexportal.org/home/) databases, including the TCGA-LUAD, GSE19188, GSE42127, GSE29016, GSE72094 cohorts and GTEx dataset. Detailed information of the GEO datasets included in this study is shown in supplemental information Table S1. Survival analysis was finished by the Kaplan–Meier plotter database (https://kmplot.com/analysis/).

#### Clinical specimens

Tissue microarrays (TMA), including 20 lung cancer specimens and 20 adjacent non- cancer specimens, were obtained between April and December of 2016 at the First Affiliated Hospital of Zhengzhou University. This study was approved by the Ethical Review Committee of the First Affiliated Hospital of Zhengzhou University.

#### Cell lines and culture

Six normal human lung and cancer cell lines–BEAS-2B, H2228, H1975, H1299, PC-9, and A549–were acquired from Fu Heng Biology (Shanghai, China). All cell lines used in this study were authenticated by short tandem repeat (STR) DNA profiling and tested for mycoplasma contamination to ensure their stability and reliability. All cells were cultured by DMEM medium (Solarbio, Beijing, China) containing 10% fetal bovine serum (VivaCell, Shanghai, China) in an atmosphere of 5% CO_2_ at 37 °C.

#### Oligonucleotides and transfection

DMT3b-specific siRNAs and HOPX-specific siRNAs were designed and synthesized to specifically knock down DNMT3B and HOPX expression, respectively. Nonsilencing siRNA oligonucleotides served as negative controls. All siRNAs were diluted in Opti-MEM and transfected into tumor cell lines using Lipofectamine 3000 following the manufacturer’s recommended protocol. Target sequences of the siRNAs are listed in supplemental information Table S2.

#### Western blot

RIPA lysis buffer containing protease inhibitors was utilized to isolate total protein from the cells. The boiled lysates were separated, and same amounts of protein were electrophoresed on 10% SDS-PAGE gels and transferred to polyvinylidene fluoride membranes (Millipore, Burlington, MA, USA). After blocking, the membranes were incubated with specific primary and secondary antibodies. Finally, the membranes were visualized using the Odyssey Infrared Imaging System (LI-COR Biosciences, Lincoln, NE, USA).

#### Immunohistochemistry

Immunohistochemistry (IHC) was finished as previously described. Based on the proportion of positive cells, the samples were scored as follows: 0, none; 1, <25%; 2, 25%–50%; 3, 51%–75%; and 4, 76%–100%. Staining intensity was assessed as follows: 0, none; 1, weak; 2, medium; and 3, strong. The total score (range 0–12) was calculated by multiplying the two sub-scores. The IHC staining index values <8 or ≥8 were identified as the cutoff values for low and high protein expression, respectively.

#### Real-time quantitative reverse transcription PCR

After extracting total RNAs, cDNA was created through reverse transcription following the protocol of the RevertAid First Strand cDNA Synthesis Kit (Thermo Fisher Scientific). Real-time quantitative PCR was accomplished by SYBR Green SuperMix (Roche, Basel, Switzerland) on a LightCycler 96. The relative quantification of the genes was determined by the 2^–ΔΔCT^ method. GAPDH was regarded as an internal control. The detailed primer sequences are listed in supplemental information Table S3.

#### Cell proliferation assay

Cell proliferation was assessed using a CCK-8 kit (Sero Biotechnology, Shanghai, China). In total, 4,000 cells were seeded into 96-well plates, and cell viability was measured using a microplate reader at 1 day, 2 days 3 days and 4days. For the clone formation assay, 500 cells/well were seeded in 12-well plates and cultured for 12–14 days and the number of colonies was determined after fixation and staining. For EdU staining assay, it was performed through EdU assay kit (RiboBio, Guanghzou, China). Briefly, cells were incubated with 50 μM EdU for 2 h, fixed with 4% paraformaldehyde for 30 min, and then stained by Apollo stain mixture and Hoechst 33342 for 30 min, respectively. Finally, A fluorescence microscope was used to take images, and the proportion of EdU-positive cells were calculated.

#### Transwell assay

The transwell assay was finished as follows: 1×10^5^ infected cells were seeded into the upper chamber in 200 μL serum-free medium, and 600 μL medium containing 10% FBS was added to the lower chamber. The cells were incubated for 36 h and then fixed and stained. Three visual fields were randomly selected and imaged using a microscope (Olympus, Tokyo, Japan).

#### Annexin V/PI staining detection

Annexin V-fluorescein isothiocyanate (FITC)/propidium iodide (PI) apoptosis detection kit were purchased from Beyotime Biotechnology (Shanghai, China). PC-9 and A549 Cells treated with si-DNMT3B or si-NC were stained according to the manufacturer’s instructions, cell apoptosis was then detected by BD FACSLyric™ Flow Cytometry System (BD Biosciences, USA).

#### Lentivirus infection and plasmid transfection

Lentiviral vectors were obtained from Hanheng Technology Corp. (Shanghai, China). A549 cells were infected with lentivirus for DNMT3B knockdown, designated as sh-DNMT3B, or control lentivirus (sh-NC). PC-9 and A549 cells were infected with lentivirus overexpressing HOPX, designated as oe-HOPX, or the corresponding negative control (vector). H2228 and H1975 cells were infected with lentivirus overexpressing DNMT3B, designated as oe-DNMT3B, or the corresponding negative control (vector). The shRNA sequences are listed in supplemental information Table S2.

#### Methylation-specific PCR

Methprimer software (http://www.urogene.org/methprimer) was utilized for the analysis of CpG island and primer design of human HOPX promoters for methylation-specific PCR (MSP). All primers used for MSP are listed in supplemental information Table S3. Briefly, DNA was extracted by Cells Genomic DNA Extraction Kit (Shanghai Acmec Biochemical Technology Co., Ltd). The concentration of DNA was measured. DNA was converted by DNA bisulfite conversion kit ( Shanghai Beyotime Biotechnology Co., Ltd). After PCR amplification, the products were analyzed on a 2% agarose gel and observed under ultraviolet light.

#### Chromatin immunoprecipitation (ChIP)

Briefly, for chromatin immunoprecipitation (ChIP), the DNA and proteins of the cells were cross-linked using 37% formaldehyde (Shanghai Macklin Biochemical Co., Ltd., Shanghai, China), and the reaction was terminated with 1.25 M glycine in a culture dish. The cells were lysed using a Cell Lysis Buffer (BPS Bioscience, San Diego, CA, USA). Ultrasound disrupti chromatin(amplitude 25–30%, 10 sec ON, 30 sec OFF,15–20 cycles). 1.5% agarose gel electrophoresis was used to verify the fragment size of chromatin (fragment size: 200 - 500bp). The immunoprecipitation of DNA fragmentation was accomplished by an immunoprecipitation kit (Beyotime Biotechnology Inc., Shanghai, China) following the manufacturer’s protocol (DNMT3B antibody concentration 1:50-1:100). Real-time PCR was performed to check DNMT3B binding sites. The primers used are listed in supplemental information Table S3.

#### Luciferase reporter assays

The wild-type or mutant HOPX promoter sequences (containing either the wild-type or mutated binding sites) were cloned into the pGL3-Basic vector. HEK293T cells were co-transfected with pGL3-HOPX-WT or pGL3-HOPX-MUT plasmid, along with either an empty vector control or oe-DNMT3B expression plasmid. After 48 hours, cells were harvested and lysed. Luciferase activity was measured using the Dual-Luciferase® Reporter Assay System (Promega) according to the manufacturer’s instructions. Firefly luciferase signals were normalized to Renilla luciferase activity. The sequences of the promoter binding sites were as follows:

Wild-type (HOPX-WT): 5’-GGGGGTGACCGTTATAAAA-3’, Mutant (HOPX-MUT): 5’-GGGGGTGACTGTTATAAAA-3’.

#### Ethics approval and consent to participate

The study had been approved by The Ethical Review Committees of the First Affiliated Hospital of Zhengzhou University (2022-KY-0640-002).

### Quantification and statistical analysis

#### Quantification

The tumor volume was measured on Day 5 and 10, and thereafter every 3 days. After 28 days, all mice were sacrificed and measured the weight and volume of the tumors.

Tumor volume was measured using a vernier caliper to determine its length and width, and tumor size was calculated and evaluated based on the tumor volume formula:

Tumor volume (mm^3^) = (width)^2^ × length/2.

#### Statistical analysis

Statistical analyses were finished through GraphPad Prism software or SPSS. The difference between two independent samples was analyzed by Student’s *t*-test or repeated-measure variance analysis. Survival curves were generated the Kaplan–Meier method. Pearson’s rank correlation test was utilized for correlation analysis, and statistical significance was set at two-sided *p* < 0.05. Data are presented as the mean ± SD of at least 3 independent experiments. ∗*p* < 0.05, ∗∗*p* < 0.01, ∗∗∗*p* < 0.001.
