# Supplementary material for: DNMT3b promotes proliferation and invasion by mediating HOPX DNA methylation in lung cancer
Source: iScience. 2025 Dec 5;29(2):114347. doi: 10.1016/j.isci.2025.114347 (PMC12876300; doi:10.1016/j.isci.2025.114347)
Supplement: Document S1. Tables S1–S3 [file mmc1.pdf]

iScience, Volume *29*

Supplemental information

DNMT3b promotes proliferation and invasion by mediating HOPX DNA methylation in lung cancer

Kelei Guan, Songfeng Zhao, Guizhen Zhang, Yun Wang, Dongdong Song, and Yanxia Ding

Supplemental information

Table S1. Gene Expression Omnibus (GEO) microarray data were used to identify the expression of DNMT3b and HOPX in tumor tissues and normal lung tissues.

Accession-number	Platform	Number of samples		Country	Years
		Non-tumor	Tumor		
GSE19188	Affymetrix	65	91	Netherlands	2010
GSE42127	Illumina	0	176	American	2013
GSE29016	Illumina	0	72	Britain	2012
GSE72094	Affymetrix	0	442	American	2015
GSE63459	Illumina	32	32	American	2015
Total		97	813		

Table S2. The siRNA/shRNA sequences of relation genes.

gene name	sequences (5'-3')
siDNMT3B#1	GGAAGGAGACCUACAUCAA<br>UUGAUGUAGGUCUCCUUCC
siDNMT3B#2	GCUUGGACUUCUACGACAA<br>UUGUCGUAGAAGUCCAAGC
siHOPX#1	CCUCAACCUCUCAAGUUCA<br>UGAACUUGAGAGGUUGAGG
siHOPX#2	GCUACAGCCUCAACAUUAA<br>UUAAUGUUGAGGCUGUAGC
siNC	UUCUCCGAACGUGUCACGUdTdT<br>ACGUGACACGUUCGGAGAAUdTdT
shDNMT3B#1	GATCCGGGAAGGAGACCTACATCAATTCAAGAGATTGATGTAGGTCTCCTTCCTTTTTG
shDNMT3B#2	GATCCGGCTGGACTTCTACGACGAACTTTCAAGAGAAGTTCGTCGTAGAAGTCCAGCTTTTTG
shNC	GCGCGATAGCGCTAATAATTTCAAGAGATTATTAGCGCTATCGCGCTTTTTT

Table S3. The primer sequences of relation genes.

gene name	Gene species	primer sequence (5'-3')
DNMT3B-F	Human	AGGGAAGACTCGATCCTCGTC
DNMT3B-R	Human	GTGTGTAGCTTAGCAGACTGG
GAPDH-F	Human	GGAGCGAGATCCCTCCAAAAT
GAPDH-R	Human	GGCTGTTGTCATACTTCTCATGG
HOPX-F	Human	GAGACCCAGGGTAGTGATTTGA
HOPX-R	Human	AAAAGTAATCGAAAGCCAAGCAC
HOPX-M-F	Human	TTTGTTGGTTTGTAGTATAAATCGG
HOPX-M-R	Human	TTTCCCACACATATTTTATAACCGT
HOPX-U-F	Human	GTGTATTTGTTGGTTTGTAGTATAAATTG
HOPX-U-R	Human	CTTTCCCACACATATTTTATAACCAT
HOPX Promter-F	Human	CCTCTGCTTCCTGCTCTCCT
HOPX Promter-R	Human	GCTTCTCCCTGCTTCTCCTC
HOPX Upstream-F	Human	GAGGAGGAGGTTGAGGAGGA
HOPX Upstream-R	Human	GCTGCTCCTGCTCTGCTTCT
